# Dual Epigenetic and Chaperone Inhibition Disrupts Hypoxia Signaling and Tumor Progression in 3-D Models of Triple-Negative Breast Cancer

**DOI:** 10.21203/rs.3.rs-10319464/v1

**Published:** 2026-07-20

**Authors:** Meenal Datta, Golnaz Asaadi Tehrani, Maksym Zarodniuk, Rebecca Kubick2, Ian Mersich, Andrew Gutierrez, Terin D’Amico, Hailey Sallaberry, Bradley Smith, Aktar Ali, Brian Blagg

**Affiliations:** Notre Dame University; Notre Dame University; University of Notre Dame; University of Notre Dame; University of Notre Dame; University of Notre Dame; University of Notre Dame; University of Notre Dame; University of Notre Dame; University of Notre Dame; University of Notre Dame

**Keywords:** HDAC inhibitors, HSP90β inhibitor, TRAP1 inhibitor, mammospheres, endothelial cells, CUT&RUN, HIF-1α

## Abstract

Therapeutic resistance remains a major barrier to treating aggressive breast cancers, particularly triple-negative breast cancer (TNBC), in which hypoxia-associated stress adaptation within the tumor microenvironment limits treatment efficacy. Here, we investigated a multi-targeted therapeutic strategy combining histone deacetylase (HDAC) inhibition with blockade of hypoxia-associated chaperones HSP90β and mitochondrial TRAP1 in 3-D breast cancer models grown from TNBC human cells. HDAC inhibitors (HDACi; vorinostat [SAHA; pan-HDACi], valproic acid [VPA; HDAC1i], and CAY10603 [CAY; HDAC6i]; showed greater efficacy than paclitaxel at reducing viability and mammosphere formation in 3-D cultures, and induced apoptosis and G2/M cell cycle arrest in TNBC cells. Combined inhibition of HDACs with our novel agents targeting HSP90β (NDNB-25) or TRAP1 (NDNT-34) synergistically reduced mammosphere viability and disrupted spheroid architecture. Mechanistically, HSP90β and TRAP1 inhibition attenuated hypoxia-associated adaptive signaling by suppressing HIF-1α, VEGFA, HSP90β, and TRAP1 gene and protein expression. In co-cultures with HUVEC cells, these effects were accompanied by impaired endothelial network formation, tumor cell migration and invasion, mitotic progression, and clonogenic growth. Following Cleavage Under Targets and Release Using Nuclease (CUT&RUN) analysis, genome-wide HIF-1α occupancy analysis revealed extensive reprogramming of HIF-1α-associated regulatory programs under HDAC, HSP90β, and TRAP1 inhibition, including pathways linked to innate immunity, interferon signaling, redox balance, autophagy, mitochondrial function, and metabolic adaptation. Together, these findings identify hypoxia-associated stress adaptation as a therapeutically actionable vulnerability in aggressive breast cancer and support coordinated targeting of epigenetic regulation, proteostasis, and mitochondrial stress adaptation to enhance treatment response.

## Inroduction

Breast cancer remains the most frequently diagnosed malignancy in women, with an estimated 321,910 cases of invasive disease and 60,730 cases of ductal carcinoma in situ (DCIS) projected in the United States in 2026 (1, 2). Among breast cancer subtypes, TNBC accounts for approximately 12–24% of cases and contributes disproportionately to breast cancer mortality due to its aggressive biology and limited availability of targeted treatment options (3, 4). Therefore, there is a critical need for rationally designed therapeutic approaches to improve treatment response in TNBC (5).

Epigenetic dysregulation plays a critical role in the progression of many cancers, including TNBC (6). Histone deacetylase inhibitors (HDACis) are potent anti-cancer agents that increase histone acetylation, promote tumor suppressor gene expression (7, 8), and modulate transcriptional programs involved in cell cycle regulation, apoptosis, differentiation, angiogenesis, and tumor-microenvironment interactions (9–11). Several HDAC inhibitors, including vorinostat, romidepsin, belinostat, and panobinostat, have been approved for hematologic malignancies (12, 13) or are being investigated in solid tumors, including TNBC **(Supplementary Table S1)** (14, 15). However, despite their broad biological activity, HDAC inhibitor monotherapy has shown limited efficacy in solid tumors including TNBC, likely because of incomplete drug penetration, tumor heterogeneity, and adaptive resistance mechanisms within the tumor microenvironment (11, 16). These limitations have prompted the development of combination strategies that combine HDAC inhibitors with chemotherapy, radiation, other targeted therapies, and immunotherapy to improve antitumor efficacy through additive or synergistic effects (17–19).

Among the adaptive mechanisms that may limit HDAC inhibitor efficacy in solid tumors, hypoxia-associated stress adaptation is particularly relevant to TNBC. Hypoxic tumor regions stabilize hypoxia-inducible factor-1α (HIF-1α), which, in turn, activates transcriptional programs that promote angiogenesis, metabolic reprogramming, invasion, stem-like tumor cell states, and survival under therapeutic stress. One such hypoxia-associated factor is tumor necrosis factor receptor-associated protein 1 (TRAP1), a mitochondrial chaperone implicated in metabolic stress adaptation. HIF-1α can induce TRAP1 expression, while TRAP1 can support HIF-1α stability and mitochondrial adaptation, forming a stress-adaptive signaling axis that may reinforce tumor survival under hypoxia (20–22). In parallel, hypoxia can engage HSP90-dependent stress responses (23). HSP90, a major ATP-dependent molecular chaperone predominantly localized in the cytosol, is upregulated under cellular stress and plays a central role in maintaining protein homeostasis by stabilizing nascent and stress-denatured proteins (24, 25). Its chaperone activity supports folding and conformational stability of HIF-1α (26), HDAC6 (27), as well as other client proteins involved in oncogenic processes (28). Together, these observations suggest that hypoxic breast cancer cells may rely on coordinated mitochondrial and cytosolic chaperone programs to sustain survival and therapeutic resistance. Based on this rationale, we hypothesized that selective of HSP90β/TRAP1 could potentiate HDAC inhibitor response in 3-D breast cancer models.

Here, we demonstrate that HDACis (vorinostat [SAHA; pan-HDACi], valproic acid [VPA; HDAC1i], and CAY10603 [CAY; HDAC6i]; are more effective than standard-of-care paclitaxel in reducing viability and mammosphere formation in 3-D breast cancer cultures, while also inducing apoptosis and G2/M cell-cycle arrest in human TNBC cells. However, the persistence of residual treatment tolerant cell populations indicated that adaptive resistance mechanisms continued to limit efficacy of HDAC inhibition. Concurrent inhibition of HSP90β or TRAP1 with our novel in-house inhibitors enhanced HDAC inhibitor response, reduced hypoxia-associated signaling, impaired endothelial network formation in angiogenic co-culture systems, and suppressed tumor cell migration, invasion, mitotic progression, and clonogenic growth. Finally, CUT&RUN-enabled genome-wide HIF-1α occupancy analysis revealed that HDAC, HSP90β, and TRAP1 inhibition reshaped HIF-1α-associated regulatory programs, including pathways linked to innate immunity, interferon signaling, redox homeostasis, autophagy, mitochondrial function, and metabolic adaptation under hypoxia. Collectively, these findings identify hypoxia-associated stress adaptation as a therapeutically actionable vulnerability in aggressive breast cancer and support a multi-layered therapeutic strategy in which epigenetic modulation combined with inhibition of hypoxia-associated chaperones overcomes resistance mechanisms.

## Methods

### Cell lines, cell culture and drug treatment

MDA-MB-231 cells were provided by Dr. Christopher Patzke, while MDA-MB-468, MCF-7, and MCF10A cells were kindly provided by Dr. Suzanne Shaw. MDA-MB-231 and MDA-MB-468 cells (aggressive, metastatic human TNBC cell lines) were used as TNBC models, whereas MCF-7 cells served as an estrogen receptor-positive (ER+) breast cancer control cell line. MCF10A cells were used as a non-tumorigenic human mammary epithelial cell control for toxicity studies. MDA-MB-231 cells were maintained in Dulbecco’s Modified Eagle Medium (DMEM), MDA-MB-468 cells in DMEM/F12, MCF-7 cells in RPMI-1640, and MCF10A cells in DMEM/F12 supplemented with 5% horse serum, 20 ng/mL epidermal growth factor (EGF), 0.5 μg/mL hydrocortisone, 100 ng/mL cholera toxin, and 10 μg/mL insulin. Culture media were supplemented with 1% penicillin-streptomycin (100 U/mL penicillin and 0.1 mg/mL streptomycin). DMEM was additionally supplemented with 4 mM L-glutamine. Cells were maintained at 37°C in a humidified atmosphere containing 5% CO_2_ and were routinely propagated as monolayers in 75 cm^2^ tissue culture flasks using 0.25% trypsin-EDTA for subculturing. For three-dimensional (3-D) culture experiments, cells were grown as multicellular spheroids/mammospheres under ultra-low attachment conditions in serum-free mammosphere culture medium consisting of DMEM/F12 supplemented with B27 supplement (1×), recombinant human epidermal growth factor (EGF; 20 ng/mL), basic fibroblast growth factor (bFGF; 20 ng/mL), and 1% penicillin-streptomycin.

Chaperone inhibitors, including NDNB-25 (HSP90β inhibitor) and NDNT-34 (TRAP1 inhibitor), were synthesized in-house. HDAC inhibitors (CAY10603, SAHA, and VPA) were purchased from Selleckchem (Selleck Chemicals, Houston, TX, USA).

#### Cell viability assay

The cell viability assay was performed using MTT. Briefly, cell cultures were treated with different concentrations of the drugs (SAHA: 0.5, 1, 2.0, 5.0, or 10.0 μM; CAY10603: 0.1, 1.0, 2.0, 5.0, or 10.0 μM; and VPA: 0.01, 0.1, 1.0, 4.0, or 10.0 mM) or control medium for 24 and 48 h, respectively. After incubation, the viability of the cells was assessed by MTT assay (Abcam, Cat# ab211091, Waltham, MA, USA) according to the manufacturer’s protocol. After 24 or 48 hours of treatment, 50 μL of serum-free media and 50 μL of the MTT reagent were added to each well. The cells were incubated at 37°C for an additional 3 hours. At the end of the specified incubation period, 100 μL of MTT solvent was added to each well. To solubilize the MTT-formazan crystals, the plate was gently agitated on an orbital shaker for 15 minutes. Absorbance was then measured at 570 nm using a Spark^®^ Multimode Microplate Reader (Tecan Group Ltd., Männedorf, Switzerland). The cell viability of, MDA-MB-231, MDA-MB-468, MCF-7 and MCF10A cells cultured in the presence of the assessed compounds was calculated as a percentage of the control cells, and the IC50 values were obtained from dose–response curves. All experiments were performed in triplicate.

#### Annexin V binding assay and cell cycle analysis by flow cytometry

MDA-MB-231 cells were seeded in 6-well plates at a density of 1×10^5^ cells/well and treated with HDACis, alongside untreated control. After 48 hours of incubation, cell death was assessed based on membrane integrity loss, indicated by high propidium iodide (PI) fluorescence following treatment with PI solution (Sigma-Aldrich, Cat# P4170, St. Louis, MO, USA). Phosphatidylserine externalization was measured using the Annexin V-FITC/PI Apoptosis Detection Kit (Invitrogen CellEvent^™^, Cat# C10493, Waltham, MA, USA), and samples were analyzed by flow cytometry. Cells were categorized into four groups based on staining: viable (Annexin V^−^/PI^−^), early apoptotic (Annexin V^+^/PI^−^), late apoptotic (Annexin V^+^/PI^+^), and necrotic (Annexin V^−^/PI^+^).

Cell proliferation was assessed using PI staining, as PI binding to DNA is proportional to the DNA content, enabling determination of the cell cycle distribution. MDA-MB-231 cells were seeded in 6-well plates at a density of 1×10^6^ cells/well and incubated overnight in complete medium. After washing with PBS, cells were treated with the respective drugs for 48 hours. Cells were then fixed in 70% ethanol for 18 hours at 4°C, followed by staining with 500 μL of PI solution containing RNase (Cell Cycle Assay Kit; Elabscience, Cat# E-CK-A351, Houston, TX, USA) in the dark at room temperature for 20 minutes. Flow cytometry was performed using a BD FACS Melody^™^ Cell Sorter (BD Biosciences, San Jose, CA, USA). All experiments were conducted in triplicate.

#### Fluorescent imaging of 3-D cultures (mammospheres)

Fluorescent imaging was performed on spheroids derived from MDA-MB-231 and MCF-7 cells following treatment with HSP90 and TRAP1 inhibitors, as well as hypoxia probe labeling. For viability assessment, spheroids were stained with Calcein AM (Thermo Fisher Scientific, Cat# C3099) and propidium iodide (PI) and imaged using a BZ-X810 widefield fluorescence microscope (Keyence, Itasca, IL, USA) equipped with a 10× objective under identical exposure settings across all experimental groups. The calibrated pixel-to-micron ratio was 0.65 μm/pixel. For each condition, three independent biological replicates were analyzed, with 10–15 spheroids (regions of interest, ROIs) per replicate imaged across 3–5 randomly selected fields of view. Image analysis was performed using FIJI/ImageJ, where dead/live cell ratios were quantified based on Calcein AM (live) and PI (dead) fluorescence intensity, and morphological parameters including spheroid area, circularity, and solidity were extracted using standardized segmentation workflows.

Fluorescent hypoxia imaging was performed using the hypoxia probe Q2N2G (MW 888.3227 g/mol; synthesized in-house). Spheroids were incubated with 10 μM probe diluted in culture medium for 4 h under standard culture conditions, followed by fixation with paraformaldehyde (PFA) prior to imaging.

#### Reverse transcription polymerase chain reaction (RT-PCR)

##### Reverse transcription polymerase chain reaction (RT-PCR)

Total RNA was isolated using TRIzol reagent (Zymo Research) according to the manufacturer’s instructions. Total RNA (500 ng) was reverse-transcribed into cDNA using the High-Capacity cDNA Reverse Transcription Kit (Thermo Fisher Scientific, Cat# 4368814). Gene expression was analyzed using TaqMan Gene Expression Assays (Thermo Fisher Scientific) for HIF1A (Hs00153153_m1), HIF2A/EPAS1 (Hs01026149_m1), HSP90β (Hs00607336_gH), TRAP1 (Hs00214715_m1), and GAPDH (Hs02758991_g1). The raw qPCR data were analyzed using the qPCR Design and Analysis app (Thermo Fisher Scientific). Outlier technical replicates resulting from clear experimental errors (e.g., pipetting inaccuracies) were excluded prior to analysis.

### Western blotting

After 24 hours of drug treatment, the cells were lysed with radio-immunoprecipitation assay (RIPA) buffer (Sigma-Aldrich, Cat# R0278, St. Louis, MO, USA) containing a protease inhibitor cocktail (Sigma-Aldrich, Cat# P8340) and 17.4 μg/mL phenylmethylsulfonyl fluoride (PMSF) (Sigma-Aldrich, Cat# P7626) for 30 minutes at 4°C. Cell lysates were centrifuged at 4°C for 20 minutes at 14,000 rpm to remove unbroken cells and organelles. The concentrations of proteins in the clarified samples were determined using the bicinchoninic acid (BCA) protein assay method (Thermo Fisher Scientific, Cat# 23225, Waltham, MA, USA). The protein samples were then analyzed by western blot using 7.5–12% sodium dodecyl sulfate-polyacrylamide gel electrophoresis (SDS-PAGE). Equal concentrations of protein were loaded into each well, which was verified later by measurement of GAPDH levels. Following electrophoresis, proteins were transferred to nitrocellulose membranes, which were blocked using 5% nonfat dry milk and then probed with specific antibodies: HIF-1α (Novus Biologicals, Cat# NB100–479, Centennial, CO, USA), HIF-2α (Novus Biologicals, Cat# NB100–122, Centennial, CO, USA), HSP90β (Cell Signaling Technology, Cat# 5087, Danvers, MA, USA), TRAP1 (Abcam, Cat# ab109633, Cambridge, MA, USA), VEGFA (Abcam, Cat# ab46154, Cambridge, MA, USA and GAPDH (Thermo Fisher Scientific, Cat# AM4300, Waltham, MA, USA). Detection of specific protein bands on the membranes was achieved by incubating the membranes in a solution containing LumiGLO Reserve chemiluminescent substrate (KPL, Cat# 54-71-03, Milford, MA, USA). Densitometric analyses were performed using the ImageJ program (National Institutes of Health, Bethesda, MD, USA).

#### Migration, clonogenic and tube formation assays

For migration, a scratch-wound assay was utilized. MDA-MB-231 and MCF-7 cells (2×10^6^) were seeded onto 6 well plates and incubated overnight. Once all the cells were attached, horizontal lines were scraped onto the bottom of each well, using a 200 μl yellow pipette tip. The cells were then washed with phosphate-buffered saline (PBS) and incubated overnight with serum-free medium. Various concentrations of NDNB-25, NDNT-34 and combinational therapy were then added to each well with 2% DMEM. Each line was photographed with a microscope video system after different time intervals, using a magnification of 10×.

The clonogenic assay was performed using crystal violet staining. MDA-MB-231 cells were adjusted to a density of 10^3^ cells/per well and incubated in a 12-well plate for 24 h. The cells were treated with SAHA μM), CAY10603(1.97 μM), NDNB-25 (500 nM), NDNT-34 (10 μM) and NDNB-25/NDNT-34 (250 nM and 5 μM), or control medium for 24 h and the medium was replaced every three days. After 18 days, colonies were fixed in PFA for 10 min and stained with 5% crystal violet for 30 min at room temperature. After washing five times with PBS and air dried the plates, colonies (≥ 50 cells/colony) were counted using an inverted microscope (Nikon, Tokyo, Japan).

Matrigel basement membrane matrix (BD Biosciences) was diluted with DMEM medium and coated in 96-well plates at 37°C for 1 h. Then, 5 × 10^4^ HUVECs were seeded in the presence of VEGFA. The tube formation ability of HUVECs was measured at 6 h. In inhibitor experiments, HUVECs were treated with SAHA, CAY10603, NDNB-25, NDNT-34 and NDNB-25/NDNT-34 for 24 h and after incubation, the number of tubes and nodes of the tubular structures were quantified using ImageJ.

#### CUT&RUN library preparation and sequencing

CUT&RUN was performed to assess genome-wide HIF-1α chromatin binding in MDA-MB-231 triple-negative breast cancer cells cultured under normoxic or hypoxic conditions and treated with HDAC inhibitors (SAHA, CAY10603, VPA) or HSP90β (NDNB-25) and TRAP1 (NDNT-34) inhibitors. Unfixed, permeabilized cells were processed using a CUT&RUN protocol adapted from (29, 30). Briefly, 500,000 cells per condition (biological duplicates) were immobilized on ConA-coated magnetic beads (Bangs Laboratories) and incubated overnight at 4°C with a CUT&RUN grade HIF-1α antibody (Abcam, ab2185) or IgG control (Invitrogen, 10500C). Antibody-bound chromatin was digested using Protein A–MNase (Addgene #123461), and cleavage was activated by calcium addition. Released DNA fragments were purified by DNA extraction kit (Cell Signaling Technology, Cat# 14209) and used for library preparation with the NEBNext Ultra II DNA Library Prep Kit for Illumina. Libraries were pooled and sequenced using paired-end 50-bp reads on an Illumina HiSeq platform.

### CUT&RUN data analysis

CUT&RUN data were processed using nf-core/cutandrun v3.2.2-g6e1125d with Nextflow v25.04.7, using Singularity containers, CPM normalization, SEACR and MACS2 peak calling, and the GRCh37 reference genome. Functional enrichment of CUT&RUN peak sets was performed with chipenrich in R. Enrichment was run separately for each sample with chipenrich using the hg19 genome build, Gene Ontology Biological Process gene sets (genesets = “GOBP”), the nearest_tss locus definition, and the chipenrich statistical method.

### Cell line datasets and HDAC sensitivity classification

Breast cancer cell lines were analyzed using DepMap CRISPR (gene effect) and RNAi (gene dependency) datasets together with DepMap PRISM drug screening profiles. To quantify genetic dependence on HDACs, dependency values for HDAC family genes were z-score normalized per gene across all breast cancer cell lines, and gene-wise z-scores were averaged within each cell line to generate a composite HDAC dependency score. In parallel, pharmacologic sensitivity was summarized from PRISM by calculating the median sensitivity metric across annotated HDAC inhibitors for each breast cancer cell line, yielding a composite HDAC inhibitor response score. Cell lines were classified as HDAC inhibition sensitive or HDAC inhibition resistant based on these composite metrics (using the pre-defined grouping strategy applied in this study).

### Differential expression analysis to define resistance-associated genes

To identify transcriptional programs associated with HDAC inhibition resistance, gene expression profiles were compared between resistant and sensitive breast cancer cell lines. Differential expression analysis was performed between groups, and genes significantly upregulated in resistant lines relative to sensitive lines were defined as the resistance-associated upregulated DEG set. This gene set was used for downstream functional enrichment analysis.

### Pathway enrichment and redundancy pruning

Pathway enrichment was performed on the upregulated DEG set using pathway gene sets (EnrichR; Reactome pathways). Enriched pathways were ranked by enrichment score (as reported by the enrichment tool). To improve interpretability and avoid inflating related pathway families, enriched terms were pruned for redundancy by collapsing near-duplicate or parent/child pathway terms and retaining only the highest-scoring representative term per family. Both the non-redundant retained pathways and the filtered redundant pathways were recorded for visualization and reporting.

### Statistical analysis

Statistical analyses and data visualization were done using GraphPad Prism (GraphPad Inc., San Diego, CA, USA).

For cell viability, each comparison was made using the Mann-Whitney test. For MTT data, comparisons were made using a 2-way analysis of variance (ANOVA). For RT-qPCR data, asterisks indicate statistical significance adjusting for multiple comparisons via multiple unpaired t-tests. Error bars represent standard error of the mean and asterisks indicate statistical significance (*p ≤ 0.05, **p ≤ 0.01, ***p ≤ 0.001, ****p ≤ 0.0001).

## Results

### HDAC Inhibitors Reduce 3-D Mammosphere Growth and Reveal Resistance Programs Linked to Proteostasis and Mitochondrial Function.

**Figure 1:**
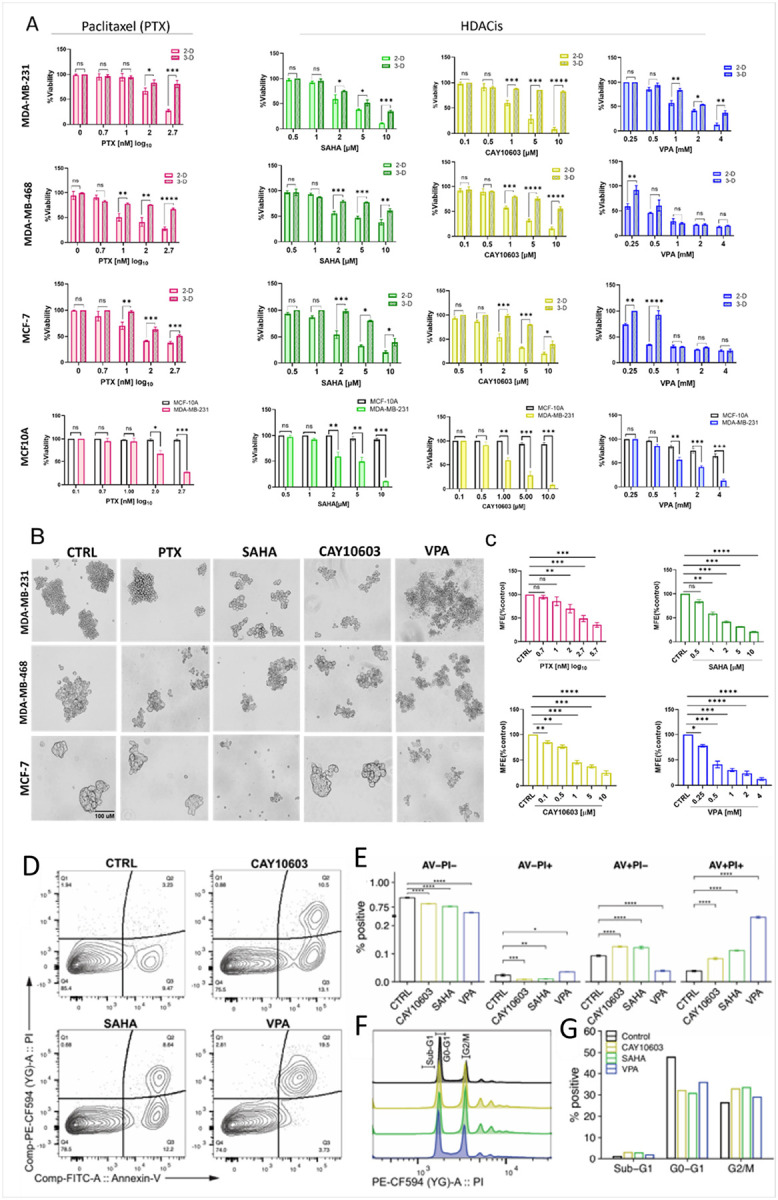
HDACis, reduce breast cancer cell viability in 2D and alter 3-D mammospheres and trigger apoptosis and cell cycle arrest. **A.** MDA-MB-231, MDA-MB468, MCF-7 and MCF10A cells were treated with SAHA (0.5–10 μM), CAY10603 (0.5–10 μM), and VPA (0.5–4 mM), in 2-D monolayers and 3-D cultures for 48 h, and subsequently viability was evaluated by MTT assay. Drug sensitivity was assessed as IC_50_. **B, C.** Evaluation of morphological changes and quantification of mammosphere formation efficacy (MFE) of breast cancer cells after 48 hr. of treatment with drug IC_50_ concentrations (PTX [36.47 nM], SAHA [3.81 μM], CAY10603 [1.97 μM], and VPA [0.26 mM]) compared to control (CTRL) as observed via representative images of phase contrast microscopy. **C.** Mammosphere formation quantification following increasing concentrations of HDACis and PTX. **D.** MDA-MB231 cells were treated with SAHA (3.81 μM), CAY10603 (1.97 μM) and VPA (0.26 mM) for 48 h, and early and late apoptosis were detected using Annexin V/PI double staining, as analyzed by flow cytometry (early = Annexin V+/PI-; late = Annexin V+/PI+). **E.** Percent of total population in each gate (early = Annexin V+/PI-; late = Annexin V+/PI+, nuclei/debris = Annexin V-/PI+, non-apoptotic = Annexin V-/PI-) was evaluated across triplicates. **F, G.** Cell cycle was analyzed by PI staining and flow cytometry. Sub-G1, G0/G1, S and G2/M indicate different cell cycle phases, as represented by separate peaks of PI fluorescence intensity. Data is presented as mean + S.E.M. from three independent experiments with triplicates. * = p<0.05; ** p<0.01; ***p<0.001 vs control via Student’s t-tests or multiple t-tests with Bonferroni correction (ns = not significant).

Understanding therapy resistance requires experimental models that better recapitulate the three-dimensional nature of tumors. We therefore compared the effects of paclitaxel (PTX) and histone deacetylase inhibitors (HDACis) on breast cancer cell growth/viability in both 2-D monolayer and 3-D culture systems (mammospheres). Human MDA-MB-231 (TNBC), MDA-MB-468 (TNBC), MCF-7 (ER+, as another breast cancer subtype control), and the non-tumorigenic MCF10A (healthy control) cells were treated with increasing concentrations of PTX, SAHA (pan-HDACi), CAY10603 (HDAC6i), or VPA; HDAC1i). In 2-D cultures, increasing drug concentrations produced dose-dependent reductions in cell viability, whereas 3-D spheroids exhibited greater resistance to treatment (Fig. 1A), highlighting the protective effect of tumor-recapitulating architecture. Among the HDAC inhibitors, valproic acid (VPA) significantly reduced cell viability in MDA-MB-468 and MCF-7 cells, while SAHA induced approximately 50% reduction of viability in both MDA-MB-231 and MCF-7 cells. CAY10603 also produced an approximately 50% reduction of viability in MCF-7 cells. Overall, HDACis demonstrated greater efficacy than PTX in 3-D mammospheres. The half-maximal inhibitory concentration (IC_50_) values in all three cells lines after 24 and 48 hours of treatment are summarized in **Table 1.**

We next evaluated the effect of the PTX and HDACis on mammosphere formation efficacy (MFE), a functional readout of stem-like growth capacity. Mammospheres larger than 60 μm were generated from MCF-7, MDA-MB-231, and MDA-MB-468 cells after 7 days in the cancer stem-like cell (CSC)-specific culture medium (Fig. 1B). MFE was quantified by counting mammospheres across passages 1–3 (P1–P3, respectively). VPA most potently impaired mammosphere formation, followed by SAHA, CAY10603, and PTX, with MCF7 displaying the highest baseline mammosphere-forming efficiency. Notably, TNBC cell lines (MDA-MB-231 and MDA-MB-468) exhibited generally higher baseline mammosphere-forming capacity compared with MCF-7, consistent with their more aggressive and stem-like phenotype, although they remained responsive to HDACi- and PTX-mediated suppression of mammosphere formation. (Fig. 1C). These findings suggest that HDAC inhibition targets both bulk tumor cells and the therapy-resistant stem-like compartment.

To determine whether the antiproliferative effect of HDACs is associated with apoptosis, we performed cell cycle analysis via Annexin V/PI flow cytometric assays in MDA-MB-231 cells treated with IC_50_ concentrations of SAHA, CAY10603, and VPA. After 48 hours of treatment, SAHA and CAY10603 predominantly induced early apoptosis, whereas VPA favored late apoptosis (Fig. 1D, E). Cell cycle analysis further demonstrated that all treatments caused G2/M arrest without substantially affecting the G0/G1 population (Fig. 1F, G). Together, these results suggest that HDAC inhibitors reduce tumor cell viability and mammosphere formation by promoting apoptosis and cell cycle blockade.

Although HDAC inhibitors showed greater efficacy than paclitaxel in 3-D breast cancer cultures, treatment did not completely abrogate tumor cell viability nor mammosphere formation, suggesting that adaptive resistance programs may persist. To investigate molecular mechanisms that may underlie incomplete HDAC inhibitor response, we leveraged integrated DepMap genetic dependency and PRISM drug response metrics to stratify breast cancer cell lines as either HDAC inhibition–sensitive or –resistant **(Fig. S1A).** After redundancy pruning, resistant-line DEGs were enriched for translation/RNA biogenesis programs and proteostasis-linked pathways, together with stress-adaptive survival signaling and genome maintenance modules **(Fig. S1B)**. Consistent with these transcriptional programs, RNAi dependency analysis (DEMETER2) identified genes selectively required in HDAC-resistant lines **(Fig. S1C)**, and enrichment of these dependencies highlighted mitochondrial proteostasis/respiration, RNA metabolism and ribosome-linked processes, and chromatin/transcription plus cell-cycle and DNA maintenance circuitry (**Fig. S1D)**. These results suggest that HDAC-resistant states may be supported in part by adaptive survival programs involving proteostasis, mitochondrial function, and increased translational activity. We therefore hypothesized that efficacy of HDAC inhibition could be enhanced by co-targeting hypoxia-associated proteostasis and mitochondrial stress pathways.

### Co-targeting HDAC and HSP90β or TRAP1 Enhances Cytotoxicity and Reduces Hypoxia in 3-D Mammospheres.

**Figure 2:**
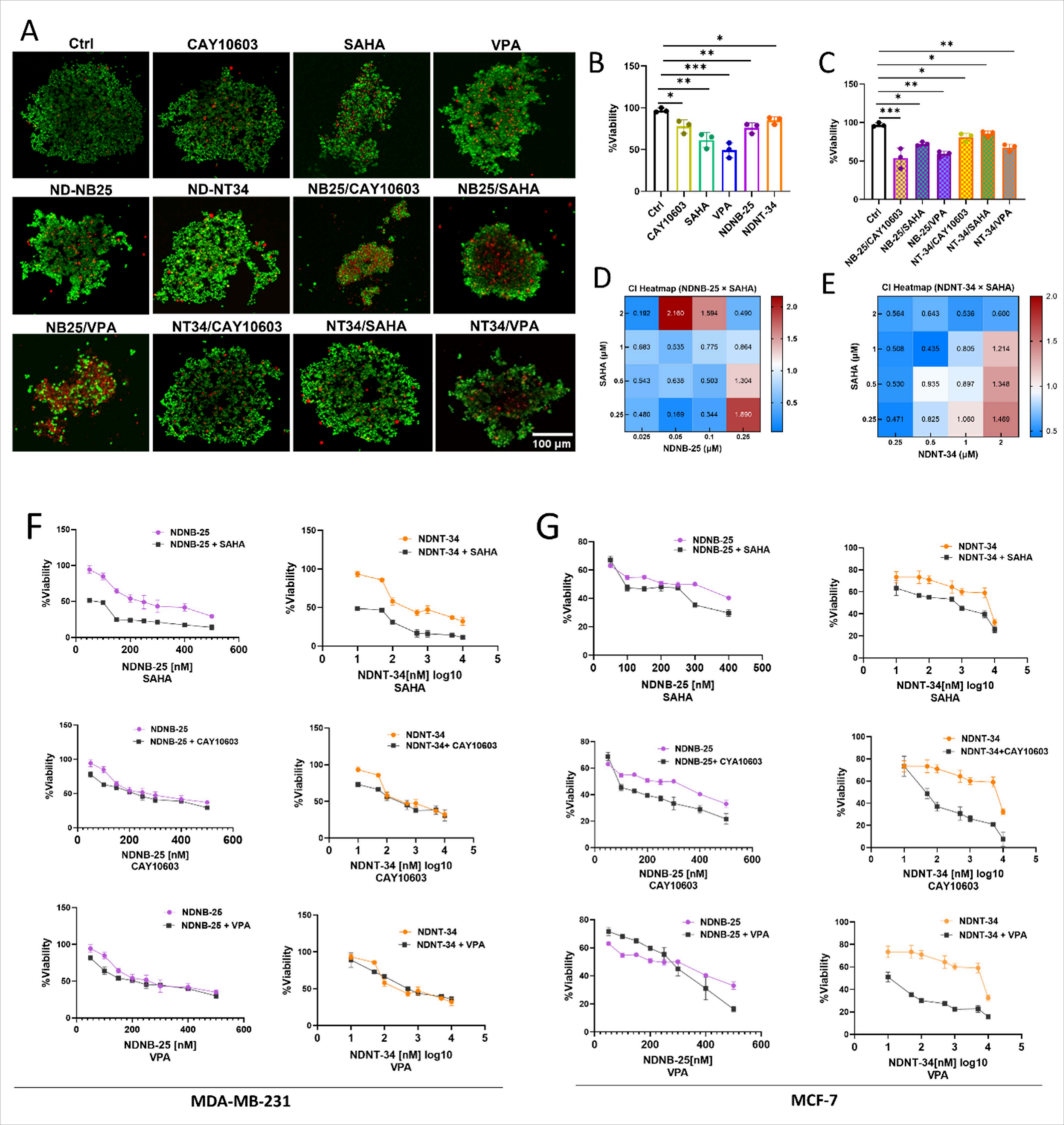
Monotherapy and combination effects of HDAC inhibitors with HSP90β and TRAP1 inhibitors. **A.** Representative images showing morphological and viability changes in breast cancer mammospheres following treatment with HDAC inhibitors (CAY10603, SAHA, and VPA), HSP90β inhibitor (NDNB-25), TRAP1 inhibitor (NDNT-34), and their combinations in TNBC cell line. **B, C.** Cell viability analysis for combinations of CAY10603 (1.97 μM), SAHA (3.81 μM), and VPA (2.02 mM) with NDNB-25 (257.5 nM) or NDNT-34 (3.46 μM) in MDA-MB-231 cell line. Bars represent mean ± SD. Statistical significance was determined relative to the untreated control group, as indicated by connecting lines. *P < 0.05, **P < 0.01, ***P < 0.001, ****P < 0.0001. **D, E.** Drug synergy analysis of NDNB-25 or NDNT-34 with SAHA. Synergy landscape generated from the dose–response matrix illustrating regions of synergistic and antagonistic interaction. **F.** MDA-MB-231 and MCF-7 cells were treated with NDNB-25 (257.5 nM) or NDNT-34 (3.46 μM) combined with SAHA (3.7 μM), CAY10603 (3.2 μM), and VPA (2.2 mM) in 2D monolayer cultures for 48 h, compared to vehicle-treated conditions (0.01% DMSO in cell culture media). Effects of drug combinations on cell viability were assessed by MTT assay.

Based on the enrichment of proteostasis and mitochondrial stress-adaptation programs in HDAC-resistant states, we focused on HSP90β and TRAP1 as therapeutically targetable components of hypoxia-associated survival pathways. HSP90β supports proteostasis by stabilizing stress-responsive client proteins (31), (including HIF-1α (PMID 33664459), whereas TRAP1 regulates mitochondrial stress adaptation and metabolic resilience under hypoxic conditions (PMID 28405578). Therefore, we evaluated whether pharmacologic inhibition of HSP90β and TRAP1 could potentiate the effects of HDAC inhibition in breast cancer cells and 3-D mammospheres. Specifically, we tested two newly synthesized in-house inhibitors, the HSP90β inhibitor (HSP90βi) (32–35). NDNB-25 and the TRAP1 inhibitor (TRAP1i) NDNT-34, in MDA-MB-231 and MCF-7 breast cancer cell lines, as well as in their derived mammospheres **(Table 2).** Combination treatment with HDAC inhibitors and either NDNB-25 or NDNT-34 significantly impaired mammosphere viability and 3-D architecture and fidelity, with the CAY10603/NDNB-25 and SAHA/NDNB-25 combinations showing pronounced reductions in viability, solidity, and circularity (Fig. 2A–C, S2A-C). Combination index analysis further showed synergistic interactions between HDAC inhibitors and NDNB-25 or NDNT-34 in both TNBC cell lines, with SAHA/NDNB-25 and SAHA/NDNT-34 showing particularly strong combinatorial effects (Fig. 2D, E). The observed dose-dependent interactions indicate that the extent of synergy is influenced by the relative concentrations of both agents. For the NDNT-34/SAHA combination, synergy was greatest at higher dose ranges, whereas lower concentrations resulted in weaker synergistic effects or antagonism. In contrast, the NDNB-25/SAHA combination exhibited maximal synergy at higher NDNB-25 concentrations and intermediate SAHA concentrations, suggesting that the optimal therapeutic interaction occurs within a specific concentration window (Fig. 2F).

To further interrogate the impact of HSP90β and TRAP1 inhibition within the 3-D tumor context, we examined cell viability and hypoxia distribution in mammospheres. Live/dead staining using calcein-AM and propidium iodide revealed that treatment with NDNB-25 or NDNT-34 monotherapies led to extensive cell death, particularly within the mammosphere core, whereas combined treatment resulted in a more uniform distribution of cell death throughout the mammosphere **(Fig. S3A-D)**. Consistent with these observations, *in situ* hypoxia imaging using our novel nitroreductase (NTR)-sensitive molecular probe Q2N2G (36, 37), a sensitive hypoxia marker in 3-D models as we have shown in other cancers, demonstrated that highly metastatic MDA-MB-231 mammospheres exhibited elevated hypoxia signal relative to MCF-7 cells, and that treatment with NDNB-25, NDNT-34, or their combination significantly reduced hypoxia-associated fluorescent signal in both models **(Fig. S3E- G)**. These findings suggest that targeting the HSP90β–TRAP1 axis reduces both mammosphere viability and hypoxia-associated signaling in 3-D breast cancer models, supporting chaperone targeting as a complementary strategy to enhance HDAC inhibitor response. Having established that HSP90β and TRAP1 inhibition can enhance HDAC inhibitor response in 3-D mammospheres, we next sought to define the downstream molecular and phenotypic effects of targeting the HSP90β-TRAP1 axis.

### HSP90 and TRAP1 Inhibition Suppresses Hypoxia-Associated Gene and Protein Expression and Disrupts Vascular Network Formation.

**Figure 3:**
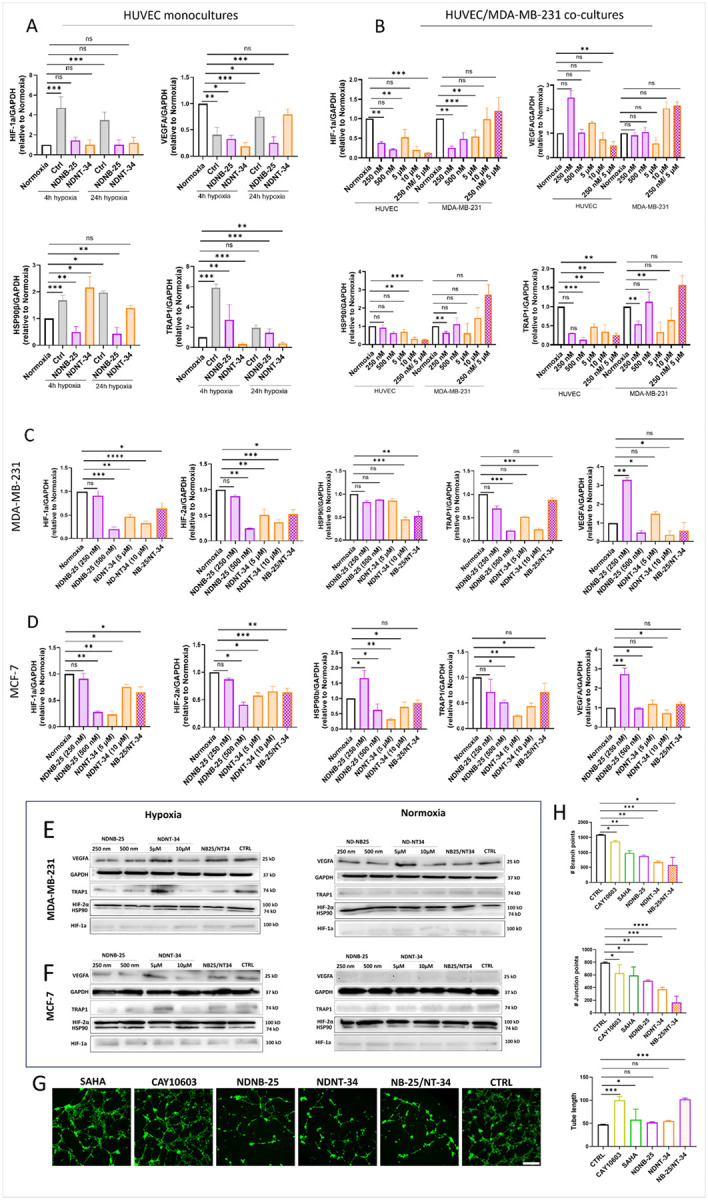
HSP90β and TRAP1 inhibition suppresses angiogenic responses under hypoxic conditions. **A, B.** RT–qPCR analysis of angiogenesis- and hypoxia-related gene expression in human umbilical vein endothelial cells (HUVECs) following 4 and 24 h of hypoxic exposure, and in MDA-MB-231/HUVEC co-culture systems after 48 h of treatment with NDNB-25 (250 or 500 nM), NDNT-34 (5 or 10 μM), or their combination under normoxic and hypoxic conditions. Data represents the mean of three technical replicates from three independent biological experiments. **C, D.** mRNA expression levels of HIF1A, HIF2A, HSP90AB1, TRAP1, and VEGFA, measured by real-time quantitative PCR following 48 h treatment with NDNB-25 (250 and 500 nM), NDNT-34 (5 and 10 μM), or the combination of NDNB-25/NDNT-34 in MDA-MB-231 and MCF-7 cell lines. **E, F.** Western blot analysis of hypoxia pathway–related protein expression in the indicated cell lines after 48 h treatment with HSP90 and TRAP1 inhibitors. **G, H.** In vitro angiogenesis (i.e., tube formation) assay evaluating endothelial tube formation following treatment with CAY10603 (5 μM), SAHA (3 μM), NDNB-25 (250 nM), NDNT-34 (5 μM), combined NDNB-25/NDNT-34, or vehicle control. Representative images were acquired at ×4 magnification and scale bar is equal to 20 uM Quantitative analysis included total tube length (pixels), number of branch points, and junction points. All experiments were performed in triplicate, and data are presented as mean ± SD. Statistical significance was determined using Student’s t-test (*P < 0.05, **P < 0.01, **P < 0.001).

We next asked whether reduction in hypoxia following HSP90β and TRAP1 inhibition was accompanied by suppression of hypoxia-responsive molecular programs, including angiogenesis. We first examined hypoxia-associated gene expression in endothelial monocultures and tumor–endothelial co-cultures. In HUVEC monocultures, hypoxia increased the expression of genes associated with hypoxia signaling, angiogenesis, proteostasis, and mitochondrial stress adaptaion, including *HIF1A*, *HSP90AB1*, and *TRAP1*. Treatment with NDNB-25 or NDNT-34 attenuated several components of this response, most notably reducing *HIF1A*, *VEGFA*, and *TRAP1* expression, although *HSP90AB1* regulation was time- and inhibitor-dependent (Fig. 3A). In MDA-MB-231/HUVEC co-cultures, treatment effects were more context-dependent, with stronger suppression of hypoxia-associated genes in HUVECs than in MDA-MB-231 cells (Fig. 3B).

We next evaluated whether these effects extended to breast cancer cells by measuring hypoxia-associated gene expression in MDA-MB-231 and MCF-7 cells following treatment with NDNB-25 and NDNT-34. After 48 hours of treatment, NDNB-25 and NDNT-34 significantly reduced *HIF1A* and *HIF2A* levels in MDA-MB-231 and MCF-7 cells. At higher drug concentrations, *TRAP1*, *VEGFA*, and *HSP90AB1* were also markedly downregulated in MDA-MB-231 cells, whereas MCF-7 cells showed broader sensitivity across lower and higher concentrations (Fig. 3C, D).

Consistent with the gene-expression results, western blot analysis showed reduced expression of hypoxia-associated proteins following NDNB-25 and NDNT-34 treatment under hypoxic conditions. HIF-1α, HSP90β, TRAP1, and VEGFA protein levels were decreased in MDA-MB-231 and MCF-7 cells after treatment, supporting the conclusion that HSP90β and TRAP1 inhibition suppresses hypoxia-associated signaling at both the RNA and protein levels (Fig. 3E, F).

Finally, we tested whether these molecular changes were accompanied by altered endothelial network formation. In HUVEC tube formation assays, NDNB-25 and NDNT-34 significantly reduced the number of branches and junctions, with combination treatment producing the strongest inhibition (Fig. 3G, H). Interestingly, combination treatment increased total tube length while reducing the number of branch and junction points, indicating formation of longer but less interconnected endothelial structures. Together, these results suggest that HSP90β and TRAP1 inhibition suppress hypoxia-associated signaling at both the transcript and protein levels, impair tumor-driven endothelial network organization, and reduce the capacity of tumor cells to promote endothelial branching and vascular network formation through paracrine tumor–endothelial interactions.

### HSP90β and TRAP1 Inhibition Reduces Tumor Cell Migration, proliferation, and Mitotic Progression.

Hypoxia stabilizes HIF-1α and activates adaptive transcriptional programs that promote tumor invasion and metastasis (38). Because HSP90β and TRAP1 inhibition reduced hypoxia-associated signaling, we next asked whether targeting this axis also restrains tumor cell motility and invasiveness.

**Figure 4:**
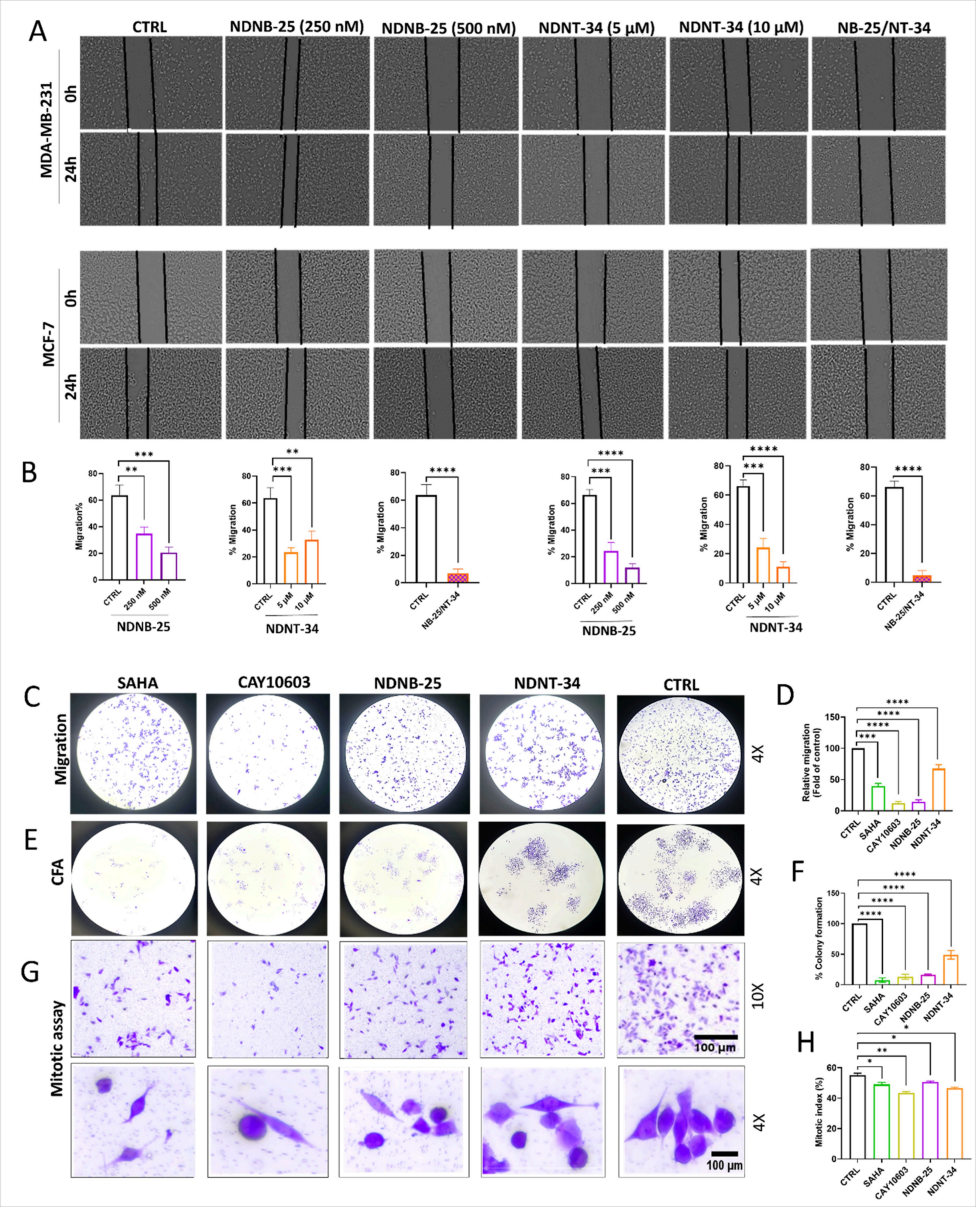
Effects of HDAC, HSP90, and TRAP1 inhibitors on cell migration, invasion, and clonogenic potential. **A, B.** Representative wound-healing assay images of MDA-MB-231 and MCF-7 cells following treatment with NDNB-25 (250 and 500 nM), NDNT-34 (5 and 10 μM), or combined treatment (NDNB-25/NDNT-34; 250 nM and 5 μM, respectively) at 0 and 24 h. Images were acquired at ×4 magnification. **C, D.** Quantitative analysis of MDA-MB-231 cell migration following treatment with HDAC inhibitors (SAHA and CAY10603), HSP90 inhibitor (NDNB-25), and TRAP1 inhibitor (NDNT-34), assessed by crystal violet staining. **E, F.** Colony formation assay demonstrating the long-term clonogenic capacity of MDA-MB-231 cells following treatment with HDAC, HSP90, and TRAP1 inhibitors. **G, H.** Mitotic index assay showing quantification of mitotic and non-mitotic migrated cells after treatment with SAHA, CAY10603, NDNB-25, and NDNT-34, indicating inhibitor-dependent suppression of invasive cell populations. All experiments were performed in triplicate, and data are presented as mean ± SD. Statistical significance was determined using Student’s t-test (*P < 0.05, **P < 0.01, **P < 0.001).

In wound-healing assays, MCF-7 and MDA-MB-231 cells treated with NDNB-25 or NDNT-34, either alone or in combination, showed markedly delayed closure of the scratch wound, with the most pronounced inhibition observed in highly invasive MDA-MB-231 cells (Fig. 4A, B). Similarly, Matrigel migration assays demonstrated a significant reduction in invasive capacity across all treatments, with NDNT-34 achieving a 67% decrease in invading MDA-MB-231 cells (Fig. 4C, D). These results indicate that targeting the HSP90–TRAP1 axis restricts both tumor cell migration and extracellular matrix invasion, two key features of metastatic progression.

We next examined whether these phenotypic changes were accompanied by altered mitotic progression and colony-forming ability. We also observed a significant reduction in colony-forming ability in MDA-MB-231 treated with NDNB-25 or NDNT-34 (Fig. 4E, F). NDNT-34 treatment induced marked mitotic arrest in MDA-MB-231 cells, resulting in an ~ 20% reduction in mitotic cells relative to control, whereas CAY10603-mediated HDAC inhibition produced an even stronger decrease. (Fig. 4G.H). Collectively, these findings suggest that inhibition of the HSP90β-TRAP1 axis suppresses hypoxia-associated tumor aggressiveness by limiting cell migration, extracellular matrix invasion, mitotic progression, and long-term clonogenic growth.

### TRAP1 Inhibition Remodels HIF-1α Chromatin Occupancy.

**Figure 5:**
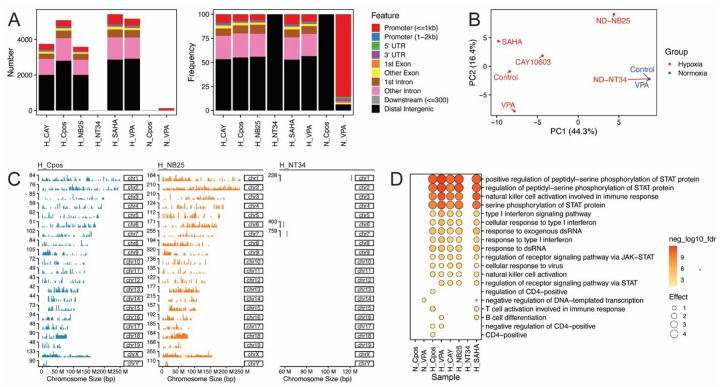
HDAC, HSP90, and TRAP1 inhibition reshapes HIF-1α chromatin binding and hypoxia-associated transcriptional programs. **A.** Genomic annotation of CUT&RUN peaks by sample. SEACR stringent peak sets from eight samples (H_CAY, CAY10603 hypoxia; H_Cpos, positive control hypoxia; H_NB25, NDNB-25 hypoxia; H_NT34, NDNT-34 hypoxia; H_SAHA, SAHA hypoxia; H_VPA, VPA hypoxia; N_Cpos, positive control normoxia; N_VPA, VPA normoxia) were annotated with ChIPseeker::annotatePeak against TxDb.Hsapiens.UCSC.hg19.knownGene using a transcription start site window of −2000 to +1000 bp. Stacked bars show the percentage of peaks assigned to each genomic feature class (left) and the corresponding estimated peak counts (right; Frequency/100 × total peaks). Features include promoter (<=1 kb and 1–2 kb), 5’ UTR, 3’ UTR, 1st Exon, Other Exon, 1st Intron, Other Intron, Downstream (<=300), and Distal Intergenic. Samples with substantial peak numbers (H_CAY, H_Cpos, H_NB25, H_SAHA, H_VPA; 3,588–5,428 peaks) were dominated by distal intergenic (52.8–56.3%) and other intronic (23.2–25.0%) annotations. In contrast, H_NT34 and N_Cpos each contained only three peaks, all annotated as distal intergenic, whereas N_VPA contained 127 peaks and was predominantly promoter-proximal (85.8% within <=1 kb of promoters). **B.**Principal component analysis of CUT&RUN samples based on SEACR consensus peak scores. Stringent SEACR peak calls from eight samples were merged into a consensus set of 159 genomic intervals, and each consensus peak was assigned the maximum overlapping score from each sample. The consensus peak-by-sample matrix was log1p transformed and analyzed by PCA after centering and scaling. Points represent individual samples, colored by hypoxia condition (Hypoxia, red; Normoxia, blue) and labeled by treatment (CAY10603, positive control, NDNB-25, NDNT-34, SAHA, VPA). PC1 and PC2 explained 44.3% and 16.4% of the variance, respectively. **C.** Genome-wide distribution of CUT&RUN peaks in the hypoxic control sample H_Cpos (left), ND-NB25-treated hypoxic sample H_NB25 (middle), and NDNT-34-treated hypoxic sample H_NT34 (right). Stringent SEACR peaks from H_Cpos (n = 5,093) were plotted across human chromosomes chr1-19, chrX, and chrY using a chromosome coverage plot weighted by the peak signal score. Each horizontal panel represents one chromosome, with genomic position on the x-axis and weighted peak coverage on the y-axis. Peaks are broadly distributed across the genome, with visible local enrichments on multiple autosomes and along chrX. **D.** GO Biological Process enrichment across CUT&RUN peak sets by chipenrich. For each sample, stringent SEACR peaks were analyzed with chipenrich using hg19, GO Biological Process gene sets (GOBP), and the nearest_tss locus definition. The dot plot summarizes GO terms with FDR < 0.05 in at least two samples. Dot size indicates the reported chipenrich effect size, and dot color indicates enrichment significance as −log10(FDR). Nineteen recurrent terms were retained, with the strongest shared enrichments concentrated in JAK-STAT/STAT and interferon signaling.

To determine how HDAC inhibition and HSP90β/TRAP1 targeting reshape hypoxia-associated gene regulatory response at the chromatin level, we profiled HIF-1α occupancy across normoxic and hypoxic conditions with and without VPA, SAHA, CAY10603, NDNB-25, and NDNT-34 treatment. Stringent peak calling identified approximately 5,000 HIF-1α peaks in hypoxia-treated samples, compared with only three peaks in normoxic cells, indicating robust hypoxia-induced HIF-1α chromatin binding (Fig. 5A). Under hypoxia, HIF-1α peaks were predominantly localized to distal intergenic and intronic regions with only a minority of peaks localized to promoter-proximal regions (Fig. 5A). This genomic distribution is consistent with enhancer-associated regulation of hypoxia-responsive transcriptional programs (2). Prominent HIF-1α peaks were observed on chromosomes 9, 18, and X and were associated with genes involved in oxygen and iron sensing (*ACO1*), autophagy and vesicular regulation (*PIK3C3*), redox homeostasis (*TXNL1*, *PRDX4*), inflammatory signaling (*CXCR1*), mitochondrial metabolism (*MAOA*), and chromatin regulation (PHF8) (**Fig. S4**).

Across hypoxic conditions with drug-treatment, HIF-1α occupancy did not globally relocalize toward promoters; instead, the genomic distribution remained largely enhancer/intron biased (Fig. 5A). By contrast, VPA treatment under normoxia produced a shift toward promoter-proximal HIF-1α occupancy, suggesting that HDAC1 inhibition specifically may alter detectable HIF-1α chromatin engagement even in the absence of hypoxic stabilization.

Principal component analysis of consensus peak signal showed that SAHA, VPA, and CAY10603 did not significantly alter HIF-1α occupancy in hypoxic cells. By contrast, NDNB-25 and NDNT-34 induced changes in HIF-1α chromatin occupancy (Fig. 5B). Notably, NDNT-34 treatment was associated with near-complete loss of detectable HIF-1α chromatin binding under hypoxia (Fig. 5C), suggesting that TRAP1 inhibition may suppress the hypoxia-induced HIF-1α regulatory program.

Pathway enrichment analysis of genes associated with HIF-1α peaks revealed recurrent enrichment for transcription start site–proximal genes involved in STAT protein phosphorylation and type I interferon signaling across hypoxic and drug-treated samples, with the exception of NDNT-34 (Fig. 5D). Together, these findings suggest that TRAP1 inhibition with NDNT-34 disrupts HIF-1α chromatin occupancy and attenuates downstream regulatory programs involving STAT and interferon-associated signaling.

## Discussion

Epigenetic dysregulation is a central feature of TNBC, and HDACis have been explored as anticancer agents due to their ability to suppress tumor cell proliferation, angiogenesis, stemness, invasion, and modulate anti-tumor immunity (14, 45). However, despite promising preclinical data, HDAC inhibitor monotherapy has shown limited efficacy (46, 47). This limitation likely reflects the ability of tumor cells to engage adaptive survival programs within complex microenvironmental contexts (48). Given their documented invasive properties (49), MDA-MB-231 cells were used as a model of aggressive TNBC to interrogate therapy-induced changes in invasion, hypoxia signaling, and microenvironmental crosstalk (20).

We recently demonstrated HDACi as a viable and potent therapeutic strategy in sensitizing glioblastoma to chemotherapy in 3-D and immune co-culture models (50). Our results show that while both PTX and HDACis exhibited potent cytotoxic effects in 2D monolayer cultures, 3-D cultured cells demonstrated significant treatment resistance, consistent with the protective effects of tumor architecture, including altered drug penetration, stem-like features, and hypoxic microenvironments. HDACis, particularly VPA, were more effective in reducing viability in 3-D mammospheres compared with PTX, consistent with prior studies showing that epigenetic modulation preferentially targets CSCs that confer drug resistance and tumor recurrence (51, 52). The differential sensitivity between 2-D and 3-D cultures underscores the importance of evaluating anticancer agents in models that mimic tumor architecture and cancer cell heterogeneity.

In terms of putative mechanisms of action, HDACis induced apoptosis and caused G2/M cell-cycle arrest in the TNBC cell line (53). SAHA and CAY10603 predominantly increased early apoptotic populations, whereas VPA induced late apoptosis, consistent with literature showing that HDACis modulate both pro- and anti-apoptotic pathways through altered expression of genes involved in programmed cell death (e.g., p21, Bax, Bcl-2) and disruption of protein chaperone complexes, TCGA and others (2, 54). HDACis, including SAHA, are known to arrest growth and induce apoptosis in diverse cancer types by reactivating silenced tumor suppressor genes and perturbing cell-cycle regulators (2, 55). Further, combined epigenetic therapy has been shown to sensitize TNBC cells to other targeted agents via enhanced histone acetylation and disruption of DNA damage responses (e.g., sensitization to PARP inhibition through “BRCAness”) (56, 57).

Despite this activity, residual mammosphere-forming populations persisted after HDAC inhibition, consistent with the limited efficacy of HDAC inhibitor monotherapy in clinical settings and suggesting that adaptive resistance programs may remain active (11, 58). Pharmacogenomic analyses supported this interpretation by identifying proteostasis, mitochondrial function, RNA metabolism, translation, chromatin regulation, and genome-maintenance pathways associated with reduced HDAC inhibitor sensitivity (59, 60). These pathways are consistent with a stress-adapted state in which residual tumor cells rely on proteostasis, mitochondrial buffering, and transcriptional plasticity to survive treatment (61, 62). This provided the rationale for targeting HSP90β and TRAP1, which regulate proteostasis and mitochondrial stress adaptation, respectively (63). Because direct inhibition of HIF-1α remains difficult due to its complex regulation and limited druggability, targeting upstream chaperone networks represents an attractive alternative (64). Selective HSP90β and TRAP1 inhibitors may provide greater therapeutic specificity and reduced toxicity compared with pan-HSP90 inhibitors while still suppressing HIF-1α-dependent adaptive responses (65).

Combination treatment with HDAC inhibitors and either HSP90β or TRAP1 inhibition enhanced cytotoxicity in breast cancer cells and 3-D mammospheres. In particular, SAHA/NDNB-25 and SAHA/NDNT-34 showed strong synergy (CI < 0.5) in both MDA-MB-231 and MCF-7 cells by combination index analysis. These results align with prior studies showing that targeting chaperone networks can sensitize cancer cells to epigenetic therapy and overcome adaptive resistance mediated by protein homeostasis mechanisms (66, 67). These effects surpass those seen with monotherapies and support the concept that multimodal targeting of proteostasis and epigenetic regulation can overcome adaptive resistance mechanisms that are otherwise maintained by molecular chaperones and epigenetic plasticity (68).

At the molecular level, HSP90β and/or TRAP1 inhibition suppressed hypoxia-associated signaling at both the RNA and protein levels (20). Our data reveal that HSP90β and TRAP1 inhibitors significantly reduced cellular hypoxia levels, as measured using NTR-reactive hypoxia probes, and downregulated hypoxia pathway genes, including *HIF1A*, *HIF2A*, *VEGFA*, *HSP90AB1*, and *TRAP1*, in breast cancer cells, endothelial cells, and tumor–endothelial co-culture systems, although responses varied by cell type, inhibitor, and dose. Western blot analysis further showed reduced HIF-1α, HSP90β, TRAP1, and VEGFA protein levels under hypoxic conditions. These findings are consistent with the known role of HSP90 chaperone activity in supporting HIF-1α stability (22, 69) and with the role of TRAP1 in mitochondrial adaptation to low oxygen (70, 71).

Angiogenic signaling is a direct downstream consequence of hypoxia-associated transcriptional programs, in part through HIF-1α-dependent regulation of VEGFA and other pro-angiogenic factors (72, 73). Consistent with the observed suppression of VEGFA, NDNB-25 and NDNT-34 altered endothelial tube formation, reduced branch and junction complexity, particularly under combination therapy. Although some treatment conditions increased total tube length, this pattern suggests formation of longer but less interconnected endothelial structures rather than productive vascular network formation. These findings complement prior studies showing that HDAC inhibitors can regulate angiogenic factors such as VEGF and disrupt vascular mimicry in TNBC models (74). Because angiogenic remodeling contributes to tumor invasion and metastasis, disruption of hypoxia-associated endothelial signaling may represent an additional mechanism by which HSP90β and TRAP1 inhibition limits aggressive tumor behavior.

Consistent with this interpretation, HSP90β and TRAP1 inhibition also reduced tumor cell migration, invasion, mitotic progression, and clonogenic growth. Wound healing assays showed delayed scratch-wound closure after NDNB-25 or NDNT-34 treatment, while Matrigel invasion assays confirmed reduced extracellular matrix invasion, particularly in MDA-MB-231 cells. These phenotypes were accompanied by reduced mitotic index and decreased colony-forming ability, suggesting that HSP90β and TRAP1 inhibition limits both acute invasive behavior and longer-term proliferative capacity. Together, these findings support a model in which targeting hypoxia-associated chaperone pathways suppresses multiple features of aggressive tumor behavior, including endothelial network remodeling, migration, invasion, and clonogenic growth. This suggests a novel functional HSP90-TRAP1 axis in regulating metastatic traits, linking proteostasis and mitochondrial stress adaptation to tumor cell motility and extracellular matrix invasion.

Finally, chromatin-level HIF-1α chromatin-occupancy analysis showed that hypoxia-associated regulatory programs are remodeled by HDAC, HSP90β, and TRAP1 inhibition. Under hypoxia, HIF-1α peaks were predominantly localized to distal intergenic and intronic regions, consistent with enhancer-associated regulation of hypoxia-responsive transcriptional programs (2). Hypoxia-associated peaks were linked to genes involved in oxygen and iron sensing, autophagy, redox homeostasis, inflammatory signaling, mitochondrial metabolism, and chromatin regulation, supporting the idea that HIF-1α coordinates multiple stress-adaptive pathways under low-oxygen conditions.

Across hypoxic conditions under drug-treatment, HIF-1α occupancy remained largely distal/intergenic and intron-biased, indicating that these treatments did not globally relocalize HIF-1α binding toward promoter regions. In contrast, VPA treatment under normoxia produced a shift toward promoter-proximal HIF-1α-associated peaks, suggesting that HDAC inhibition may influence HIF-1α-associated chromatin accessibility independent of hypoxic stabilization. Conversely, the selective HDAC inhibitor CAY10603 induced minimal redistribution, whereas HSP90β and TRAP1 inhibition attenuated hypoxia-associated HIF-1α binding at metabolic, redox, and autophagy-related loci, consistent with the role of HSP90/TRAP1 in stabilizing HIF-1α and supporting mitochondrial adaptation under hypoxia (75, 76). Functional enrichment analyses further demonstrated that HIF-1α–associated regions were linked to stress-adaptive and inflammatory signaling pathways, including, IL6/JAK/STAT3, angiogenesis, ROS and PI3K/AKT/mTOR signaling, aligning with emerging evidence that hypoxia intersects with epigenetic regulation to reprogram tumor–immune crosstalk in TNBC (77, 78). Chromosome-level analysis identified hypoxia-enriched HIF-1α binding at loci regulating oxygen sensing, redox homeostasis, and metabolic adaptation, consistent with established roles of these genes in hypoxia-driven tumor survival and invasion (79). Enrichment at MAOA and the epigenetic co-activator PHF8 further supports coordinated regulation of mitochondrial metabolism and canonical HIF-1α–dependent transcriptional programs, including *VEGFA* and *GLUT1* (80). Collectively, these findings demonstrate that HDAC inhibition and hypoxia cooperatively reprogram HIF-1α chromatin engagement, reinforcing the concept that epigenetic therapies can inadvertently potentiate hypoxia-driven transcriptional networks and immune-modulatory pathways in TNBC.

In summary, our results across multiple TNBC models indicate that aberrant activation of the hypoxia pathway can be mitigated by HSP90/TRAP1 inhibition, thereby enhancing the therapeutic efficacy of HDAC inhibitors. Given that both HDAC inhibitors and chaperone-targeting agents are currently in clinical development, the findings from this study suggest that a combination of the two agents may provide additional clinical benefit in the treatment of TNBC. Future studies employing *in vivo* models, patient-derived xenografts, and correlative clinical biomarkers will be essential to validate these mechanisms and translate this approach into effective therapies for patients with therapy-resistant, hypoxia-driven TNBC.

## Supplementary Material

Supplementary Files

This is a list of supplementary files associated with this preprint. Click to download.

- Table1.xlsx
- Table2.xlsx
- FigS1.png.jpg
- fulllengthuncroppedoriginalwesternblots.docx
- FigS3.png.png
- Table3.xlsx
- FigS2.png.png
- FigS4.png.png

Tables are available in the Supplementary Files section.

## Figures and Tables

**Table:** 

HDAC inhibitor	HDAC class/target	TNBC setting	Combination therapy	Phase	Key finding/status	Trial ID	Reference
Entinostat (MS-275)	Class I HDACi	Metastatic/advanced HER2 − breast cancer including TNBC cohorts	Entinostat + nivolumab + ipilimumab	Phase I	Immune checkpoint combination; evaluated safety, dose, and immune activation in HER2 − disease including TNBC subset	NCT02453620	(39)
Romidepsin	Class I HDACi	Metastatic TNBC / BRCA-associated breast cancer	Romidepsin + cisplatin ± nivolumab	Phase I	Early-phase study showing limited monotherapy activity; explored synergy with DNA-damaging agents and immunotherapy	NCT02393794	(40)
Belinostat	Pan-HDACi	Metastatic TNBC	Belinostat + ribociclib (CDK4/6 inhibitor)	Phase I	Preclinical rationale supported CDK/HDAC synergy; early clinical evaluation of cell-cycle + epigenetic targeting	NCT04315233	(41)
Vorinostat (SAHA)	Pan-HDACi	TNBC / HER2 − breast cancer (neoadjuvant settings)	Vorinostat + carboplatin + nabpaclitaxel	Phase I/II	Increased histone acetylation; limited clinical response but acceptable safety in combination regimens	NCT00616967	(42)
Tucidinostat (Chidamide)	Class I HDACi	Metastatic TNBC	Tucidinostat + capecitabine	Phase II	Improved response signals in epigenetically responsive subgroups; ongoing evaluation in TNBC	NCT05390476	(43)
Vorinostat (SAHA)	Pan-HDACi	Relapsed/metastatic TNBC	Vorinostat + PARP inhibitor (olaparib)	Phase I	Preclinical synergy via DNA damage + chromatin remodeling; early clinical translation ongoing	NCT03742245	(44)
